# Children born very preterm experience altered cortical expansion over the first decade of life

**DOI:** 10.1093/braincomms/fcae318

**Published:** 2024-09-17

**Authors:** Lisa S Gorham, Aidan R Latham, Dimitrios Alexopoulos, Jeanette K Kenley, Emily Iannopollo, Rachel E Lean, David Loseille, Tara A Smyser, Jeffrey J Neil, Cynthia E Rogers, Christopher D Smyser, Kara Garcia

**Affiliations:** Department of Psychiatry, Washington University School of Medicine, St. Louis, MO 63110, USA; Department of Neurology, Washington University School of Medicine, St. Louis, MO 63110, USA; Department of Neurology, Washington University School of Medicine, St. Louis, MO 63110, USA; Department of Neurology, Washington University School of Medicine, St. Louis, MO 63110, USA; Department of Neurology, Washington University School of Medicine, St. Louis, MO 63110, USA; Department of Psychiatry, Washington University School of Medicine, St. Louis, MO 63110, USA; Department of Neurology, Washington University School of Medicine, St. Louis, MO 63110, USA; Department of Psychiatry, Washington University School of Medicine, St. Louis, MO 63110, USA; Department of Neurology, Washington University School of Medicine, St. Louis, MO 63110, USA; Department of Pediatrics, Washington University School of Medicine, St. Louis, MO 63110, USA; Department of Radiology, Washington University School of Medicine, St. Louis, MO 63110, USA; Department of Psychiatry, Washington University School of Medicine, St. Louis, MO 63110, USA; Department of Pediatrics, Washington University School of Medicine, St. Louis, MO 63110, USA; Department of Neurology, Washington University School of Medicine, St. Louis, MO 63110, USA; Department of Pediatrics, Washington University School of Medicine, St. Louis, MO 63110, USA; Department of Radiology, Washington University School of Medicine, St. Louis, MO 63110, USA; Department of Radiology & Imaging Sciences, Indiana University School of Medicine, Evansville, IN 46202, USA; Department of Mechanical Engineering and Materials Science, Washington University in St. Louis, St. Louis, MO 63130, USA

**Keywords:** prematurity, brain development, surface area, cortical expansion

## Abstract

The brain develops rapidly from the final trimester of gestation through childhood, with cortical surface area expanding greatly in the first decade of life. However, it is unclear exactly where and how cortical surface area changes after birth, or how prematurity affects these developmental trajectories. Fifty-two very preterm (gestational age at birth = 26 ± 1.6 weeks) and 41 full-term (gestational age at birth = 39 ± 1.2 weeks) infants were scanned using structural magnetic resonance imaging at term-equivalent age and again at 9/10 years of age. Individual cortical surface reconstructions were extracted for each scan. Infant and 9/10 cortical surfaces were aligned using anatomically constrained Multimodal Surface Matching (aMSM), a technique that allows calculation of local expansion gradients across the cortical surface for each individual subject. At the neonatal time point, very preterm infants had significantly smaller surface area than their full-term peers (*P* < 0.001), but at the age 9/10-year time point, very preterm and full-term children had comparable surface area (*P* > 0.05). Across all subjects, cortical expansion by age 9/10 years was most pronounced in frontal, temporal, and supramarginal/inferior parietal junction areas, which are key association cortices (*P*_Spin_ < 0.001). Very preterm children showed greater cortical surface area expansion between term-equivalent age and age 9/10 compared to their full-term peers in the medial and lateral frontal areas, precuneus, and middle temporal/banks of the superior sulcus junction (*P* < 0.05). Furthermore, within the very preterm group, expansion was highly variable within the orbitofrontal cortex and posterior regions of the brain. By mapping these patterns across the cortex, we identify differences in association cortices that are known to be important for executive functioning, emotion processing, and social cognition. Additional longitudinal work will be needed to understand if increased expansion in very preterm children is adaptive, or if differences persist into adulthood.

## Teaser

Gorham and Latham *et al*. report that, compared to full-term children, children born very preterm experience increased cortical expansion in association cortices from term-equivalent age to age 9/10 years.

## Graphical Abstract

**Graphical Abstract:**
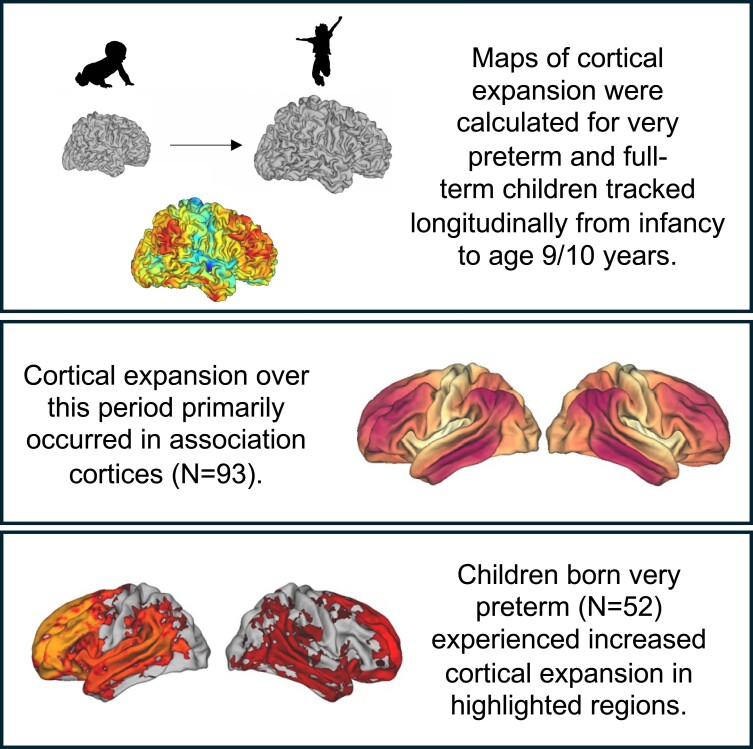

## Introduction

The brain develops rapidly between birth and adolescence. Cortical surface area nearly doubles during the first year of life and expands by another 20% in the second year.^1^ By age 6 years, brain volume reaches 90% of its adult size.^2,3^ This rapid increase in size is not only nonlinear but also non-uniform across the brain. Research dating back over 100 years indicates that the cortex can be divided into key sensorimotor and association cortices,^4,5^ and numerous neuroimaging studies have found that brain development follows a predictable pattern in which sensorimotor areas develop first and association cortices develop later.^2,6,7^ Importantly, these trajectories of brain development are sensitive to environmental influences such as exposure to poverty,^8^ and deviations in these trajectories have been linked to adverse neurodevelopmental outcomes. For example, smaller cortical surface area has been associated with lower cognitive and language scores at age 2 and decreased cortical surface area at age 10 has been linked to both lower cognitive ability and increased rates of psychiatric disorders.^9-11^ Together, current research highlights the importance of developmental trajectories in understanding the emergence of cognitive and psychiatric functioning in youth, as well as how environmental and biological factors may influence these trajectories.

The final trimester of gestation is critical for brain development. Key processes, including neuronal migration, dendritic development, sulci formation, and synaptogenesis, occur during this period^12,13^ and may be altered by preterm birth. Compared to full-term infants, preterm infants exhibit reduced whole brain, white matter, and cortical and subcortical gray matter tissue volumes,^14,15^ but thicker cortex in the somatosensory, frontal, and insular regions^16,17^ at term-equivalent age (TEA). Similarly, surface-based analyses have revealed reduced global and regional cortical surface area^14,18-20^ as well as reduced global and regional gyrification^17,19,20^ at TEA. Finally, prematurity is associated with a decrease in general cognitive functioning and working memory, as well as increased rates of inattention/hyperactivity, anxiety, social difficulties, and autism spectrum disorders.^21^ However, few longitudinal neuroimaging cohorts of very preterm (VPT) infants have been studied, highlighting the need for a deeper understanding of brain development in VPT children after TEA.

By studying a longitudinal cohort of VPT and full-term (FT) children followed from birth to age 9/10 years, the primary aim of this study was to quantify individualized cortical surface area changes from infancy through school age and investigate how prematurity alters cortical expansion during this period. We hypothesized that expansion would be greatest in late-developing association cortices for both VPT and FT individuals, and that expansion of these regions may be altered in the VPT individuals. Additionally, we hypothesized that VPT infants would exhibit decreased surface area at both TEA and at age 9/10 compared to their FT peers. Understanding these differences in cortical expansion may help explain previously established differences in neurodevelopmental outcomes and identify markers for targeted interventions designed to improve cognitive and neuropsychiatric outcomes.

## Materials and methods

### Recruitment

As part of a longitudinal study, postnatal cortical changes were examined among VPT and FT children. VPT infants were born at 23–29 weeks’ gestation and recruited from the St. Louis Children’s Hospital Level-IV Neonatal Intensive Care Unit. FT infants were born at 37–41 weeks’ gestation and recruited from the adjoining maternity hospital. FT infants were scanned using magnetic resonance imaging within the first four days of life, while VPT infants were scanned at TEA (35–40 weeks’ postmenstrual age, PMA). Eligible participants were brought back at 9/10 years of age for a subsequent MRI scan. Participants were excluded due to chromosomal abnormalities, congenital infections, and/or if the mother was unable to provide informed consent. VPT infants with Grade III/IV intraventricular hemorrhage, post-hemorrhagic hydrocephalus, cystic periventricular leukomalacia, or moderate/severe cerebellar injury were excluded from the current analysis. Additionally, FT children were excluded for positive maternal urine drug screens and/or acidosis on cord blood gas measurements.^22^ All FT children were singletons. This study was conducted in accordance with The Code of Ethics of the World Medical Association and was approved by the Washington University Human Studies Committee. Parental written informed consent was obtained for all participants. Children also provided assent at age 9/10.

### Magnetic resonance imaging

Term-equivalent images were obtained using a Siemens Trio 3T scanner (Erlangen, Germany) and included a structural T2-weighted sequence acquired with TR = 8600 ms, TE = 161 ms, and a voxel size of 1 × 1 × 1 mm^3^. Images were reviewed by a neuroradiologist (J.S.S.) and pediatric neurologist (C.D.S.) to evaluate for brain injury. Images at 9/10 years of age were obtained using a Siemens Prisma 3T scanner (Erlangen, Germany). Structural T1-weighted data were acquired with TR = 2500 ms, TE = 2.9 ms, with a voxel size of 1 × 1 × 1 mm^3^. These images were also reviewed by a neuroradiologist (J.S.S.) and pediatric neurologist (C.D.S.) for injury. All FT subjects had normal scans at this time point.

### Image quality

T2-weighted images were evaluated and scored for motion and scanner artifacts by a highly experienced data analyst (D.A.) and were categorized as having no, mild, moderate, or severe motion. Mild motion images had only a few slices in which the cortical ribbon and deep nuclear structures were not well delineated. Moderate motion typically involved many slices where the demarcation between the cortical gray matter ribbon and white matter was not easily identifiable and deep structures were poorly defined. Inclusion of these images was based on the segmentation outputs and the reliability of the manual editing of segmentations. Severe motion images were unusable and not processed. They were characterized by global motion that rendered structures unidentifiable. Only participants with images of sufficient quality at both time points were included in this longitudinal analysis.

### Cortical surface reconstruction

For the infant cortical surfaces, initial brain segmentation was performed using the surface-based Melbourne Regional Infant Brain atlas (M-CRIB-S) pipeline.^23^ After segmentation edits by a data analyst on our team with extensive experience in this population (D.A.), a neonatal surface reconstruction algorithm from the modified Medical Image Registration ToolKit (MIRTK) (https://github.com/BioMedIA/MIRTK) was used to create cortical midthickness surfaces using Connectome Workbench.^24^ For the 9/10 surfaces, Freesurfer cortical surface reconstruction methods were used to generate cortical midthickness surfaces.^25^

Cortical midthickness surfaces were used to calculate global cortical surface area of both right and left hemispheres with Connectome Workbench. The portion of the medial wall encompassing the corpus callosum, septum pellucidum, and deep nuclear gray matter was excluded from all calculations of the cortical surface area using a standardized mask.

### Surface expansion mapping

**Figure 1:**
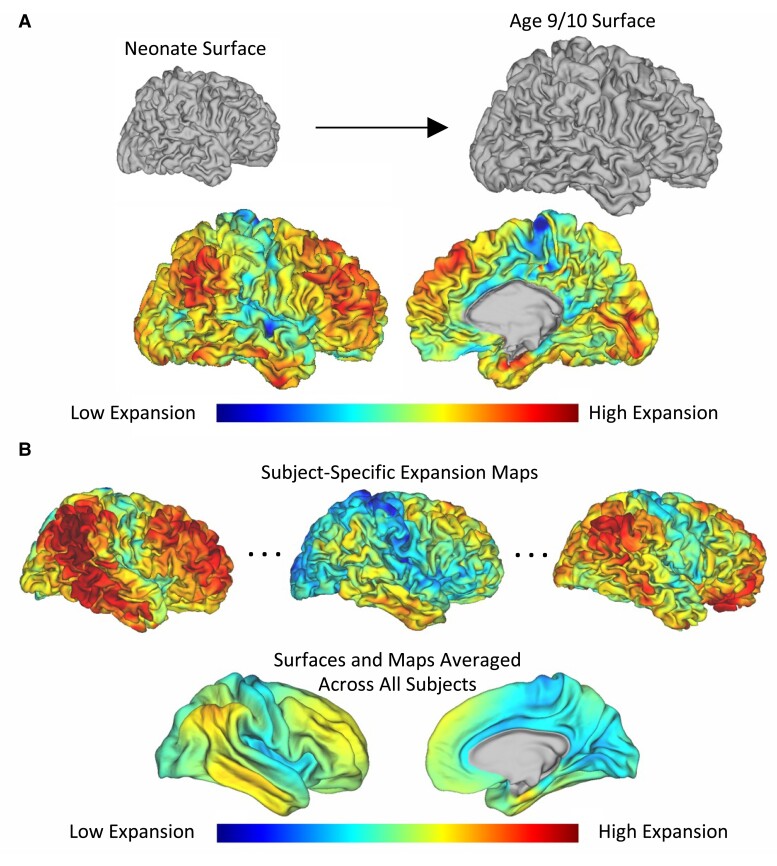
**Anatomically constrained multimodal surface matching (aMSM) can be used to calculate cortical expansion. (A)** aMSM uses a neonate surface and an age 9/10 surface to compute a map of expansion for a particular individual across the studied time window. **(B)** Subject-specific expansion maps can then be averaged across all subjects in a group to generate average maps of expansion. For both panels, expansion values range from 2 (the equivalent of the surface doubling in size) to 4 (the equivalent of the surface quadrupling in size). All brains are shown to scale.

To generate vertex-wise maps of cortical expansion for each subject, cortical surface pairs were aligned using anatomically constrained Multimodal Surface Matching (aMSM).^26,27^ First, optimized spherical projections were generated for each cortical midthickness surface in Connectome Workbench using 10 000 iterations of smoothing followed by 10 cycles of 100 iterations of inflation. To guide initial alignment, mean curvature maps were also generated from each cortical midthickness surface.^28^ Maps corresponding to each pair of surfaces were used as input and reference data, and pairs of cortical midthickness surfaces – rescaled to a common cortical surface area for internal calculations (10 000 mm^2^) – were used as input and reference anatomical surfaces.^27^ Registrations resulted in optimized point correspondence between the two surfaces at the level of the control point grid resolution such that local growth throughout the cortex could be estimated as the vertex-wise areal expansion at each control point, *i*. For each subject, point correspondence across surface pairs was manually inspected for accuracy, and expansion maps were calculated as the vertex-wise areal expansion ratio (*J_i_* = *A_2,i_/A_1,i_*) between registered surfaces at full scale (Fig. 1). As in Garcia *et al.,*^27^ unbiased registrations and expansion maps were computed from the average of registrations in each direction: the neonatal surface (input) to the 9/10 surface (reference) and 9/10 surface (input) to the neonatal surface (reference).^27^

## Statistical analysis

### Cohort demographic comparisons

We compared our VPT and FT groups on several demographic variables, which are displayed in Table 1. Gestational age at birth, PMA at infant scan, age at 9/10 scan, birthweight, and area deprivation index (ADI) percentile at birth or at age 9/10 were compared between groups using a *t*-test. Sex assigned at birth was compared between groups using a chi-squared test. Race, ethnicity, and maternal education were compared between groups using Fischer’s exact test. For all demographic comparisons, *P* < 0.05 was considered statistically significant.

**Table 1:** Demographic information

Variable	VPT Subjects (*N* = 52)	FT Subjects (*N* = 41)	*P*-Value
Gestational age at birth	26.5 weeks +/− 1.6 weeks	39.2 weeks +/− 1.2 weeks	< 0.001***
Postmenstrual age at infant scan	37.6 weeks +/− 1.5 weeks	39.2 weeks +/− 1.2 weeks	< 0.001***
Sex assigned at birth (male/female)	N = 25/27	N = 16/25	0.508
Age at 9/10 scan	9.9 years +/− 0.7 years	9.8 years +/− 0.7 years	0.760
Birthweight	916.6 grams +/− 234.3 grams	3314.6 grams +/− 443.2 grams	<0.001***
ADI National Percentile at birth	70.48 +/− 24.19	77.98 +/− 21.55	0.118
ADI National Percentile at age 9/10	64.37 +/− 26.95	76.98 +/− 24.10	0.020*
Race, % (N)	50 (26) African American	80.5 (33) African American	0.007**
	44.2 (23) White	19.5 (8) White	
	1.9 (1) Asian	0 (0) Asian	
	3.8 (2) Biracial	0 (0) Biracial	
Ethnicity, % (N)	3.8 (2) Hispanic	2.4 (1) Hispanic	1.0
	94.2 (49) Non-Hispanic	97.6 (40) Non-Hispanic	
	1.9 (1) Person Missing Data		
Number of multiples	7 Sets of twins/triplets	No twins/triplets	N/A
Maternal education, % (N)	5.8 (3) Junior High	29.3 (12) Junior High	0.002**
	25 (13) High School	41.5 (17) High School	
	38.5 (20) College	6.8 (11) College	
	11.5 (6) Graduate School	2.4 (1) Postgraduate Degree	
	1.9 (1) Postgraduate Degree		
	17.3 (9) People Missing Data		

Demographic information for the subjects included in the aMSM analyses. Data are provided as mean +/− standard deviation. *P*-values for gestational age at birth, PMA at infant scan, age at 9/10 scan, birthweight, and ADI national percentile at birth and age 9/10 come from *t*-tests between the two groups. *P*-value for sex comes from a chi-squared test. *P*-values for race, ethnicity, and maternal education come from Fischer’s exact test.

### Global statistics

To account for important covariate factors known to influence brain development, we first conducted cross-sectional multiple regression analyses of hemispheric surface areas at both the infant and age 9/10 time points. Previous studies have suggested the effects of prematurity, exposure to poverty, and sex assigned at birth on brain development.^8,14^ At the infant time point, we examined the effect of prematurity, as well as poverty at birth [defined by the national percentile of the area deprivation index (ADI), where a higher percentile indicates greater neighborhood poverty^29^], sex, and PMA at the scan on both left and right hemisphere surface areas. At age 9/10, we similarly examined the effects of prematurity, ADI at age 9/10, age at scan, and sex on both left and right hemisphere surface areas. Finally, we examined the effects of prematurity, ADI at birth, age at scan, and sex on the global cortical expansion of both left and right hemispheres from TEA to age 9/10. In these comparisons, we chose to consider the effect of ADI at birth over age 9/10 since early exposure to poverty has been strongly linked to differences in brain volumes,^30^ white matter connectivity,^31^ and cortical network development,^32^ and can shape trajectories of brain development over time.^8^ To account for multiple comparisons across left and right hemispheres, *P* < 0.025 was considered statistically significant.

### Surface statistics

To facilitate group-level comparisons, individual expansion maps were resampled and projected to the HCP 164k fsLR template space.^33^ This was accomplished using MSMSulc and 164k_fs_LR spheres output from the CIFTIFY pipeline^34^ applied to Freesurfer output from scans at 9/10 years. To visualize patterns of both average expansion and variability in expansion within each group, maps of mean and standard deviation were generated at each vertex. To compare our maps of expansion with the sensorimotor-association axis, a known framework of brain development,^7^ we conducted permutation spin tests in R Studio^35^ using the Gordon 333 parcellation scheme^36^ and 10 000 rotations. For all statistical analyses, expansion values [defining the ratio, *J*, at each vertex or region of interest (ROI)] were log-transformed as log_2_*J* to facilitate descriptions designed for normally distributed data.

The Permutation Analysis of Linear Models (PALM) suite of statistical testing^37^ was utilized to create statistical comparisons of local growth patterns and control for covariates of interest. In all cases, threshold-free cluster enhancement (TFCE) and family-wise error rate (FWER) correction were employed, assuming both exchangeable errors (to allow permutations of the input data) and independent/symmetric errors (to allow sign-flipping of the input data).^38^

To determine regions of significantly higher and lower relative expansion (higher or lower than the global expansion of that hemisphere) within the VPT and FT groups, we performed two-tailed one-sample t-tests on CIFTIFY-aligned, log-normalized maps of relative expansion, where relative expansion was defined as the true cortical expansion (*J*) divided by the total areal expansion of that hemisphere (*A_2_/A_1_*). To determine areas of statistically higher or lower growth in the VPT group as compared to the FT group, we performed a two-tailed two-sample *t*-test on log-normalized maps of true cortical expansion (*J*). Similar to comparisons of global cortical expansion, this regional comparison considered PMA at infant scan, ADI percentile at birth, age at 9/10 scan, and sex as covariates. After FWER, which accounts for multiple comparisons across vertices, *P* < 0.05 was considered statistically significant.

## Results

A total of 93 children (52 VPT and 41 FT), scanned as infants and again at 9/10 years of age, were included in the final analysis. Demographic characteristics of the sample are shown in Table 1. Clinical characteristics of the VPT children are shown in Supplementary Table 1. Information about how these 93 children were selected from the larger parent study is displayed in Supplementary Fig. 1. VPT infants had a lower PMA at infant scan (*P* < 0.001), lower birthweight (*P* < 0.001), and lower ADI percentiles (indicating less exposure to neighborhood disadvantage) at age 9/10 compared to their FT peers (*P* = 0.020). The two groups did not differ in terms of ADI percentile at birth (*P* = 0.118), age at 9/10 scan (*P* = 0.760), or sex (*P* = 0.508).

### Biological and environmental effects on hemispheric surface area at term-equivalent age and age 9/10

First, we examined the effects of prematurity, ADI, PMA at infant scan, age at 9/10 scan, and sex on hemispheric surface area at both the infant and 9/10 time points using linear models (Table 2). At TEA, prematurity (left hemisphere: *T* = −3.992, *P* < 0.001; right hemisphere: *T* = −4.064, *P* < 0.001), higher ADI percentiles (left hemisphere: *T* = −2.874, *P* = 0.005; right hemisphere: *T* = −2.482, *P* = 0.015), earlier PMA at infant scan (left hemisphere: *T* = 6.680, *P* < 0.001; right hemisphere: *T* = 6.611, *P* < 0.001), and being assigned female sex at birth (left hemisphere: *T* = −3.998, *P* < 0.001; right hemisphere: *T* = −4.071, *P* < 0.001) were associated with smaller surface area. On average, the VPT group had mean hemispheric surface areas of 29 894 mm^2^ (left) and 29 726 mm^2^ (right) on the infant scans, whereas the FT group had mean hemispheric surface areas of 34 465 mm^2^ (left) and 34 347 mm^2^ (right) on the infant scans.

**Table 2:** Surface area at term-equivalent age and 9/10 years

Surface Area at Term-Equivalent Age						
	Left Hemisphere			Right Hemisphere		
Variable	Estimate	*T*-Value	*P*-Value	Estimate	*T*-Value	*P*-Value
Prematurity	−2718.67	−3.992	<0.001***	−2762.91	−4.064	<0.001***
ADI National Percentile at Birth	−35.87	−2.874	0.005**	−30.92	−2.482	0.015*
Age at infant scan (PMA in weeks)	1459.84	6.680	<0.001***	1442.18	6.611	<0.001***
Sex (Female)	−2352.22	−3.998	<0.001***	−2390.94	−4.071	<0.001***

Surface Area at Age 9/10 Years						
	Left Hemisphere			Right Hemisphere		
Variable	Estimate	*T*-Value	*P*-Value	Estimate	*T*-Value	*P*-Value
Prematurity	−2184.35	−1.287	0.201	−2292.7	−1.345	0.182
ADI National Percentile at 9/10 years	−180.45	−5.579	<0.001***	−179.5	−5.524	<0.001***
Age at 9/10 scan (years)	−548.74	−0.471	0.639	−340.3	−0.291	0.772
Sex (female)	−6957.15	−4.160	<0.001***	−7233.9	−4.305	<0.001***

Linear models of left and right hemisphere surface areas at term-equivalent age and age 9/10, predicted by prematurity status, ADI percentile, age at scan, and sex. Each of the four models (left and right hemispheres; TEA and age 9/10) has 88 degrees of freedom. *indicates *P* < 0.025; ** indicates *P* < 0.01; *** indicates *P* < 0.001.

At age 9/10, higher ADI percentiles (left hemisphere: *T* = −5.579, *P* < 0.001; right hemisphere: *T* = −5.524, *P* < 0.001) and being assigned female at birth (left hemisphere: *T* = −4.160, *P* < 0.001; right hemisphere: *T* = −4.305, *P* < 0.001) were associated with smaller surface area, but prematurity (left hemisphere: *T* = −1.287, *P* = 0.201; right hemisphere: *T* = −1.345, *P* = 0.182) and age at 9/10 scan (left hemisphere: *T* = −0.471, *P* = 0.639; right hemisphere: *T* = −0.291, *P* = 0.772) were not related to surface area. While ADI of the FT group remained similar across the 10-year period, ADI of the VPT group decreased between infancy and age 9/10, leading to a statistically significant difference between groups by age 9/10 (*P* = 0.020, see Table 1). Regardless, at both time points, both groups were relatively disadvantaged compared to the national average. On average, the VPT group had mean hemispheric surface areas of 86 754 mm^2^ (left) and 86 967 mm^2^ (right) on the age 9/10 scans, which was similar to the FT group with mean hemispheric surface areas of 86 057 mm^2^ (left) and 86 356 mm^2^ (right) on the age 9/10 scans.

Finally, we created a global variable of cortical expansion, defined by dividing the hemispheric surface area at age 9/10 by the hemispheric surface area at the infant scan for each subject. We then used linear models to predict expansion using prematurity, ADI percentile at birth, PMA at infant scan, age at 9/10 scan, and sex as covariates. Being born very preterm was associated with increased left (*T* = 2.772, *P* = 0.007) and right (*T* = 2.591, *P* = 0.011) hemispheric expansion over the period between TEA and age 9/10. Additionally, having a lower PMA at the infant scan was associated with increased left (*T* = −6.134, *P* < 0.001) and right (*T* = −5.738, *P* < 0.001) hemispheric expansion over this period. On the other hand, ADI at birth, sex, and age at 9/10 did not significantly predict global expansion. Complete results for this analysis can be found in Supplementary Table 2.

### Expansion from infancy to age 9/10 occurs in key association cortices

**Figure 2:**
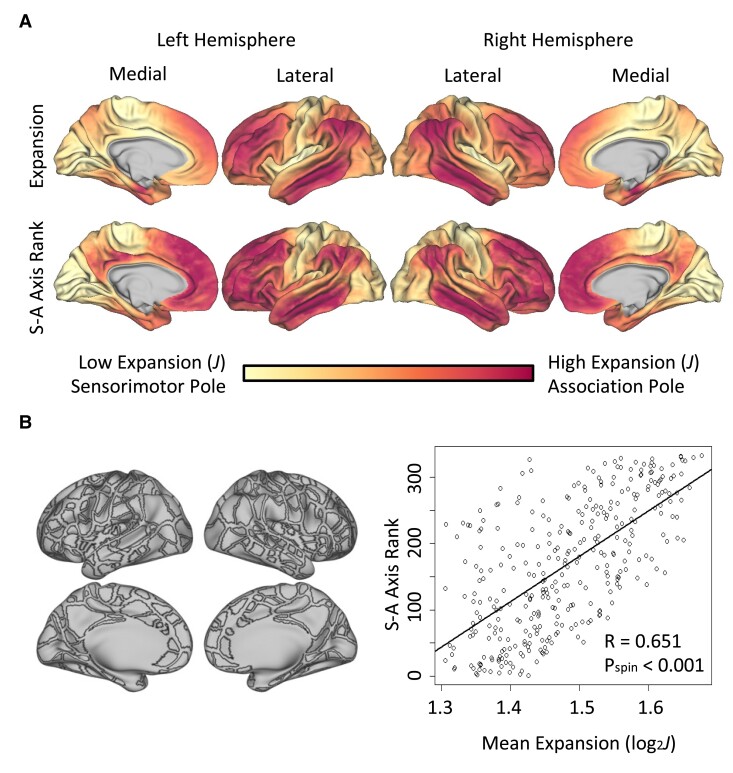
**Cortical expansion occurs in key association cortices. (A)** The top row represents average expansion across all subjects. The bottom row represents the sensorimotor-to-association axis as described in Sydnor 2021,^7^ with the lighter areas representing key sensorimotor cortices and the darker areas representing key association cortices. In both cases, color bar extremes span the 2nd to 98th percentile of individual vertex values. **(B)** The group average map of expansion was compared to the sensorimotor-association axis using a permutation spin test (right). Each dot represents a region of interest (ROI) from the Gordon 333 parcellation, and this parcellation is illustrated to the left of the scatterplot. Log-normalized expansion is shown on the *x*-axis, such that a value of 1.0 represents the surface area doubling, and an expansion value of 2.0 represents the surface area quadrupling.

Subject-specific expansion maps spanning the 10-year period were created for each subject and then averaged to explore general spatial trends (Fig. 1). Average expansion maps generated across all 93 subjects (pooling VPT and FT subjects) revealed pronounced cortical expansion in frontal, temporal, and supramarginal/inferior parietal junction areas, bearing striking similarity to the sensorimotor-association axis.^7^ To explore this quantitatively, this group average map was compared to the sensorimotor-association axis using a permutation spin test, confirming that expansion was most pronounced in key association cortices (*R* = 0.651, *P*_spin_ < 0.001; Fig. 2). Analysis separating subjects into FT and VPT groups found very similar results (Supplementary Fig. 2, *R* > 0.5 and *P*_spin_ < 0.001 in both cases).

### Prematurity is associated with increased expansion of association cortices from infancy to age 9/10

**Figure 3:**
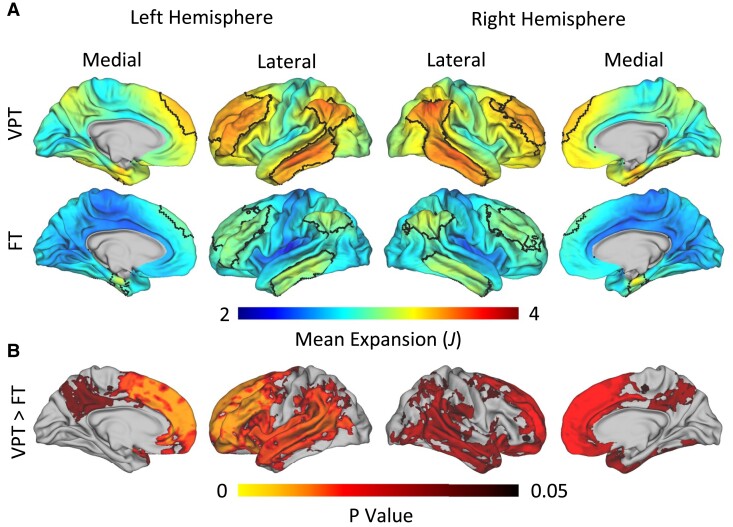
**Very preterm (VPT) subjects experience increased expansion in association regions. (A)** Average cortical expansion from birth to age 9/10 in VPT children (top row) and full-term (FT) children (middle row). Black outlines represent areas of the brain exhibiting significantly greater expansion compared to the rest of the brain. An expansion value of 2.0 represents the surface area for that region doubling, and an expansion value of 4.0 represents the surface area quadrupling. **(B)** Threshold-free cluster enhancement (TFCE)-applied family-wise error rate (FWER)-corrected *P*-values from a two-tailed *t*-test for areas where VPT children have significantly greater expansion across the 10-year period compared to FT children when controlling for postmenstrual age (PMA) at infant scan, area deprivation index (ADI) percentile at birth, age at 9/10 scan, and sex. This analysis was conducted using permutation analysis of linear models (PALM) software.

We next investigated the role of prematurity while controlling for ADI percentile at birth, sex, PMA at infant scan, and age at 9/10 scan on cortical expansion. Prematurity was associated with increased expansion in the medial and lateral frontal areas, precuneus, and middle temporal/banks of the superior sulcus junction (*P* < 0.05; Fig. 3). These areas of increased expansion were key association cortices, which were confirmed using a permutation spin test [*R* = 0.636, *P*_spin_ < 0.001 (Supplementary Fig. 3)]. Maps corresponding to the *T* statistics for this primary comparison, as well as covariate effects, are displayed in Supplementary Fig. 4. Notably, younger PMA at infant scan was strongly associated with increased expansion across nearly all vertices of the brain, suggesting that a substantial amount of cortical expansion occurs over the range of infant scan age range included (35–41 weeks PMA). Additionally, lower ADI percentile at birth (indicating less poverty) was associated with increased expansion in posterior regions, though this effect was smaller than that of prematurity. Age at 9/10 scan and sex were observed to have relatively weak effects on expansion (Supplementary Fig. 4).

Given that the VPT and FT groups differed in terms of PMA at infant scan (*P* < 0.001, Table 1), and PMA at infant scan was observed to have a strong effect on cortical expansion in most brain regions, supplementary analysis of matched groups was conducted to confirm the separate effects of prematurity and PMA at infant scan. We first split the VPT children into two groups: an older-at-scan VPT group (*N* = 21) that was matched on PMA at scan to our FT controls, and a younger-at-scan VPT group (*N* = 31) that was scanned at an earlier PMA (Supplementary Fig. 5A). Of note, the two VPT groups did differ in terms of medical risk score, as the VPT infants scanned at an older PMA were, on average, sicker than the VPT infants scanned at an earlier PMA (*T* = 2.16, *P* = 0.038). This difference is not surprising, as healthier babies were discharged from the hospital at an earlier age.

Comparisons between FT and the age-matched (older scan 1 PMA) VPT subjects revealed that the VPT subjects had increased expansion in key association cortices. Although statistical power was low for this comparison due to the smaller age-matched VPT sample, trends were strikingly similar to our main analysis, despite the fact that this only included age-matched VPT children (Supplementary Fig. 5B). Additionally, we directly compared expansion in the older-at-scan and younger-at-scan VPT groups (two-sample *t*-test). Age-at-scan differences were strikingly similar to the effect of PMA at scan in our main analysis (Supplementary Fig. 5C). Together, these results indicate that the effects of PMA at infant scan and prematurity are likely separate, despite the confound of the VPT and FT groups differing in terms of PMA at infant scan.

### Prematurity is associated with increased variability in regional expansion

**Figure 4:**
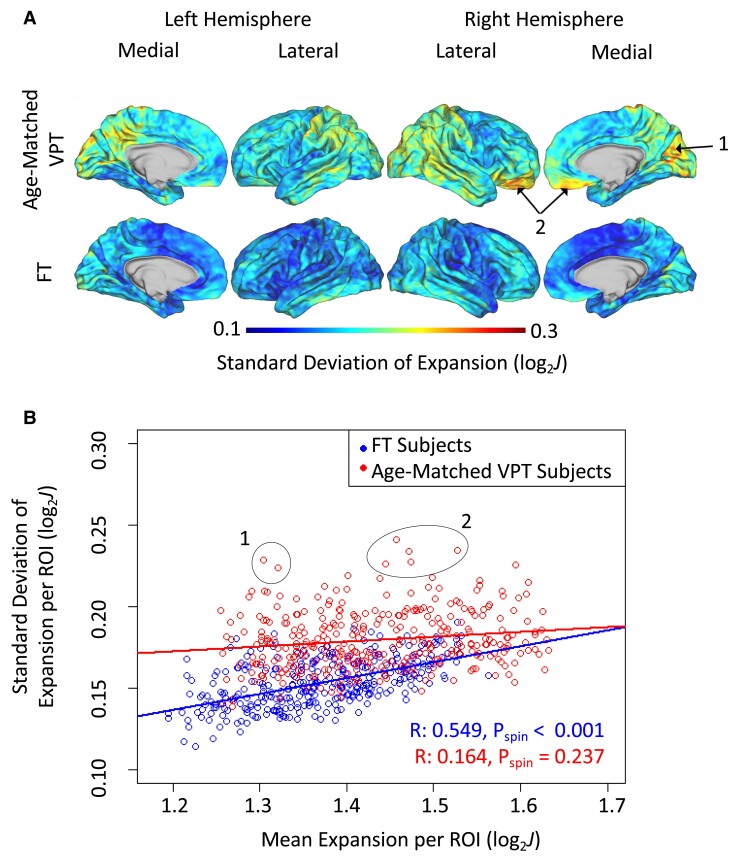
**Very preterm (VPT) subjects exhibit increased variance in expansion. (A)** Age-matched VPT subject (top row) and full-term (FT) subject (bottom row) standard deviation in log-normalized expansion. Age-matched VPT subjects had greater overall variability compared to the FT subjects. Example areas of high variability are denoted with arrows and highlighted again in part B. **(B)** Relationship between mean values of expansion and standard deviation for age-matched VPT and FT groups. Each data point represents a region of interest (ROI) from the Gordon 333 parcellation. Log-normalized expansion is shown on the *x*-axis, such that a value of 1.0 represents the surface area doubling and an expansion value of 2.0 represents the surface area quadrupling. Age-matched VPT subjects are illustrated in red, and FT subjects are illustrated in blue. Clusters labeled 1 and 2 correspond to regions of high variability in the VPT group. Approximate location of each cluster is denoted in panel **A**.

Finally, we leveraged our subject-specific maps of cortical expansion to assess variability in expansion over the period from TEA to age 9/10. As illustrated in Fig. 4A, within-group standard deviation of cortical expansion was first calculated at each vertex from subject-specific maps of cortical expansion. Comparisons displayed here focus on FT and age-matched older-at-scan VPT groups. Notably, both mean and standard deviation of expansion were higher in age-matched VPT subjects compared to the FT subjects at all vertices. Next, we conducted permutation spin tests to assess whether variability is correlated with regions of greatest expansion during this period (Fig. 4B). For the FT group, the mean and standard deviation of expansion were highly correlated (*R* = 0.549, *P*_spin_ < 0.001), with the magnitude of within-group variation increasing approximately linearly with magnitude of within-group average expansion (Fig. 4B). This correlation is not surprising, since the magnitude of variation is expected to increase with magnitude of signal. However, when comparing the mean and standard deviation of expansion within the age-matched VPT group, no correlation was observed (Fig. 4B and R = 0.164, P_spin_ = 0.237). As denoted in Fig. 4A, expansion for the VPT group was most variable within the orbitofrontal cortex and posterior regions of the brain, despite these regions experiencing only moderate expansion on average.

### Additional ad hoc sensitivity analyses

In addition to the analyses described earlier, we conducted several additional ad hoc sensitivity analyses to better interpret our results. Since the VPT group had lower ADI percentiles by age 9/10 compared to the FT group (Table 1), and seven sets of twins or triplets (compared to no multiples in the FT group), we created additional smaller samples that (i) were matched on ADI percentile at age 9/10, (ii) included only one sibling from each twin/triplet family group, and (iii) included only one sibling from each twin/triplet family group *and* were matched on ADI percentile at age 9/10. We repeated all cross-sectional analyses with these samples and found similar results in all cases (Supplementary Tables 3, 4, and 5).

Finally, given the findings of similar surface area in VPT and FT groups at age 9/10 years, we additionally analyzed the relationships between total cortical gray and white matter volumes and prematurity, ADI percentile, sex, and age at scan at both the infant and age 9/10 time points to determine if there were differences in predictors of surface area versus tissue volumes. At TEA, having a younger PMA at infant scan (*T* = 7.105, *P* < 0.001) and being female (*T* = −3.183, *P* = 0.002) were significantly associated with smaller total cortical gray matter volume, but being born preterm (*T* = −2.138, *P* = 0.035) and having a higher ADI percentile at birth were only weak predictors (*T* = −1.961, *P* = 0.053; Supplementary Table 6). Smaller cerebral white matter volume at TEA was significantly predicted by a higher ADI percentile at birth (*T* = −2.868, *P* = 0.005), a younger PMA at infant scan (*T* = 3.406, *P* < 0.001), and being female (*T* = −3.769, *P* < 0.001), but was only weakly predicted by prematurity (*T* = −1.892, *P* = 0.062) (Supplementary Table 7).

At age 9/10, smaller total cortical gray matter volume was significantly predicted by being female (*T* = −4.061, *P* < 0.001) and having a higher ADI percentile at 9/10 (*T* = −6.683, *P* < 0.001) but was only weakly predicted by prematurity (*T* = −1.974, *P* = 0.052) and was not predicted by age at 9/10 scan (*T* = −0.990, *P* = 0.325; Supplementary Table 6). Smaller total cerebral white matter volume at age 9/10 was predicted by prematurity (*T* = −3.687, *P* < 0.001), a higher ADI percentile at age 9/10 (*T* = −4.722, *P* < 0.001), and being female (*T* = −3.995, *P* < 0.001), but was not predicted by age at 9/10 scan (*T* = 0.774, *P* = 0.441; Supplementary Table 7).

## Discussion

The goal of the current study was to examine how the human cortex expands from infancy to middle childhood, as well as whether differences in cortical expansion exist between VPT and FT individuals over this period. For both VPT and full-term children, expansion was greatest in frontal, temporal, and supramarginal/inferior parietal junction areas. VPT birth was also associated with a smaller left and right hemispheric surface area at the infant scan after controlling for ADI percentile, younger PMA at the infant scan, and sex. However, in contrast to the research hypothesis, prematurity was not associated with a smaller hemispheric surface area at age 9/10. Instead, cortical expansion from term to age 9/10 was higher among VPT individuals. Specifically, VPT infants showed greater cortical expansion in the medial and lateral frontal areas, precuneus, and middle temporal/banks of the superior sulcus junction (Fig. 3). VPT infants also showed increased variation in expansion compared to their FT peers (Fig. 4). While highest variation was localized to areas of highest expansion within the FT group, this pattern was not observed within the VPT group.

In the present study, cortical expansion from the neonatal to school-age period was most pronounced in key association cortices, which is consistent with existing literature on brain development from birth to preadolescence.^7^ Cortical expansion and development have been shown to occur along a sensorimotor-to-association axis, with sensory and motor areas maturing first and association cortices associated with socioemotional, executive, and other higher-order functions developing later.^2,7,39^ This temporal pattern has been demonstrated in cohorts of multiple age ranges.^27,39^ Previous work from our group focusing on a subgroup of VPT infants from this same cohort, scanned serially during the neonatal period and analyzed using aMSM, demonstrated this temporal pattern of cortical expansion early in development from birth to TEA.^27^ Additionally, an atlas-based study comparing FT infants to healthy young adults found that parts of the frontal, parietal, and lateral temporal cortex expanded nearly twice as much as regions in the medial occipital and insular cortices during the postnatal period.^40^ Similarly, others have identified increased cortical surface expansion in the sensorimotor and insular cortices first and in high-order association subdivisions later.^6^ Finally, studies of cortical thickness have shown that cortical thinning first occurs in the primary sensory-motor cortex and eventually progresses to secondary, multimodal, and supramodal cortical areas later in adolescence.^3^ The present study suggests that VPT infants, like FT infants, have patterns of cortical expansion that generally parallel this axis during the first 10 years of life.

Key study findings also showed that VPT infants have greater cortical expansion globally and specifically within the association cortices during the first 10 years of life. The VPT subjects also had greater variability globally compared to their FT peers, with the greatest heterogeneity found in posterior regions and some areas in the orbitofrontal cortices. Given that the neonatal brain develops in a posterior to anterior fashion,^41^ VPT birth may disrupt early posterior brain development leading to greater variability in expansion in these regions among VPT children. Additionally, the orbitofrontal cortices represent a distinct area of interest, as structural^19^ and functional activation abnormalities^42-44^ have been observed among VPT infants and related to subsequent problems in executive function.^45-47^

Lastly, differences in both cross-sectional measures of surface area and in cortical surface expansion were found between VPT and FT children. Consistent with previous work from our group and others,^14,18-20^ VPT infants had a smaller surface area at term-equivalent age compared to FT infants. Current study findings extend previous work to suggest that although prematurity was associated with increased cortical expansion across the 10-year period, there were no VPT and FT between-group differences in surface area by age 9/10 years. Similar to previous research,^8^ exposure to neighborhood disadvantage was instead found to be independently associated with surface area at age 9/10. While few longitudinal neuroimaging studies of VPT children exist, our results are aligned with one study that examined cortical volumes between infancy and age 7: VPT children had reduced cortical volumes compared to their FT peers, but this was primarily due to reduced cortical thickness rather than surface area.^48^ Kelly *et al.,*^48^ as well as other studies that report smaller brain volumes, aberrant connectivity, and differences in white matter microstructure for VPT children at age 7, 12, and 13 compared to FT children,^48-52^ did not specifically look at the age 9/10 time point, an age at which surface area may be peaking.^53^

While the lack of between-group differences in surface area at age 9/10 could be interpreted as the VPT children demonstrating neurodevelopmental catch-up to their FT peers, it is also possible that the increased expansion within the VPT group could be maladaptive.^50^ In humans, cortical surface area expands rapidly in the first year of life and peaks during late childhood, followed by a decline during adolescence and young adulthood.^53^ Although speculative, our findings at age 9/10 may represent a developmental epoch in which trajectories of surface area growth and development transition from expansion to decline, and which may differ between VPT and FT children. For example, the VPT children’s age at peak surface area could be delayed, which would yield comparable surface area if the surface area of FT children has begun to decline at age 9/10.^53^ This hypothesis is partially supported by Thompson *et al*., who found that between term-equivalent age and age 7 years, very preterm children had smaller volumetric brain growth.^54^ The observed increase in expansion from infancy to 9/10 years of age may still accommodate an ultimately decreased surface area, as observed in adulthood.^53^ Future work in preterm samples with more than two time points of data will be needed to map out differences in trajectories in more detail.

### Strengths and limitations

A strength of our study is the use of aMSM, which allows the generation of individualized expansion maps for each subject. These individualized maps allow us to view subject-specific deviations from normal trajectories and quantify inter-subject variation within groups. However, limitations of the current study include our modest cohort size due to the need for high-quality surfaces at both time points. Moreover, we only had two time points of data, which limited our ability to model nonlinear patterns of cortical expansion. Additional time points throughout early and middle childhood would be useful to delineate more detailed trajectories of structural brain development between VPT and FT children. Finally, while we did control for PMA at infant scan in our analyses, we note that our VPT and FT groups were not matched on PMA at infant scan. Due to the rapid amount of brain growth that occurs in the first few weeks of life, future research should strive to use children matched on PMA at infant scans or use larger cohorts once they become available.

### Future directions

To further understand differences in developmental trajectories between VPT infants and FT infants, including whether the increased expansion in VPT infants is productive or maladaptive, future work will need to follow VPT infants through adolescence. Developmental follow-up of the study cohort is ongoing, with the adolescent wave at age 14/15 years currently underway. This additional time point of data collection will allow further investigation of whether the VPT infants continue to have similar surface areas to their full-term peers through adolescence, or if they show altered trajectories in cortical development. More importantly, we will explore whether observed differences in cortical expansion relate to psychiatric or neurodevelopmental outcomes. Additionally, future work may assess predictors of observed variability in cortical expansion within the VPT group, as well as the relationship between expansion and changes in functional or white matter connectivity over time. Finally, given our findings related to exposure to neighborhood poverty, future work with additional cohorts or with measures of individual-level socioeconomic status such as family income should be conducted to better understand the impact of social risk factors on trajectories of structural brain development.

## Conclusion

This study used aMSM, a powerful tool for mapping detailed spatiotemporal changes in cortical expansion, to show differences in structural brain development between VPT and FT infants over a 10-year period. VPT infants showed decreased surface area at TEA compared to FT infants. Over the first decade of life, VPT children exhibited increased cortical expansion in key association areas, as well as increased variability in specific regions of known vulnerability due to their early development. Future work utilizing multiple time points of longitudinal neuroimaging data in adolescence and young adulthood will be necessary to elucidate key differences in trajectories for VPT and FT children. Understanding these trajectories of brain development is critical as it may help explain differences in neurodevelopmental outcomes for VPT individuals and inform the development and implementation of targeted interventions.

## Supplementary Material

fcae318_Supplementary_Data

## Data Availability

Data will be available upon request with a data use agreement.
