# Supplementary material for: Children born very preterm experience altered cortical expansion over the first decade of life
Source: Brain Commun. 2024 Sep 17;6(5):fcae318. doi: 10.1093/braincomms/fcae318 (PMC11426356; doi:10.1093/braincomms/fcae318)
Supplement: fcae318_Supplementary_Data [file fcae318_supplementary_data.pdf]

SUPPLEMENTARY MATERIALS

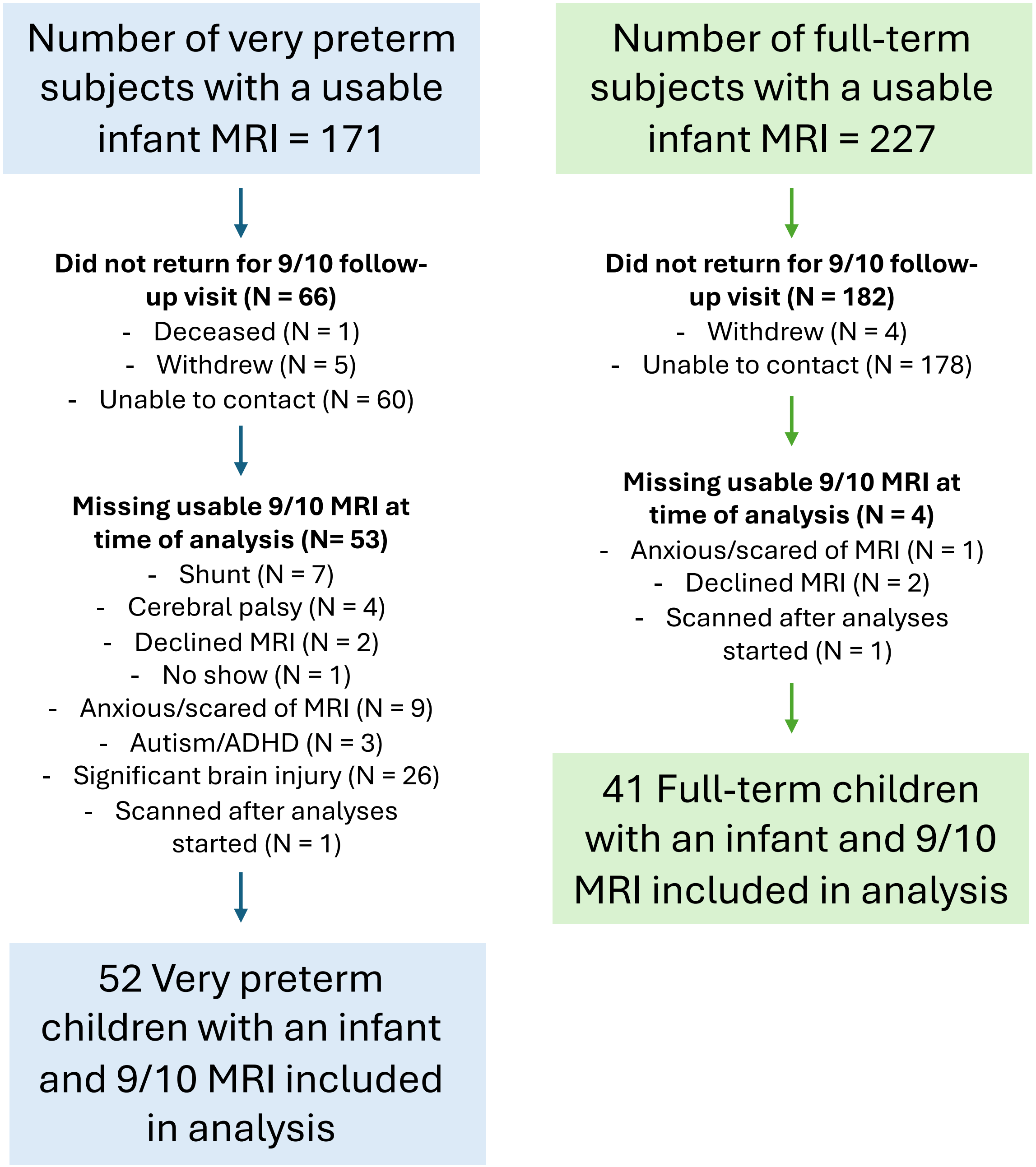

Supplementary Figure 1 Subject retention flowchart. This chart depicts the reasons why subjects of the broader parent study were excluded from the current analyses.

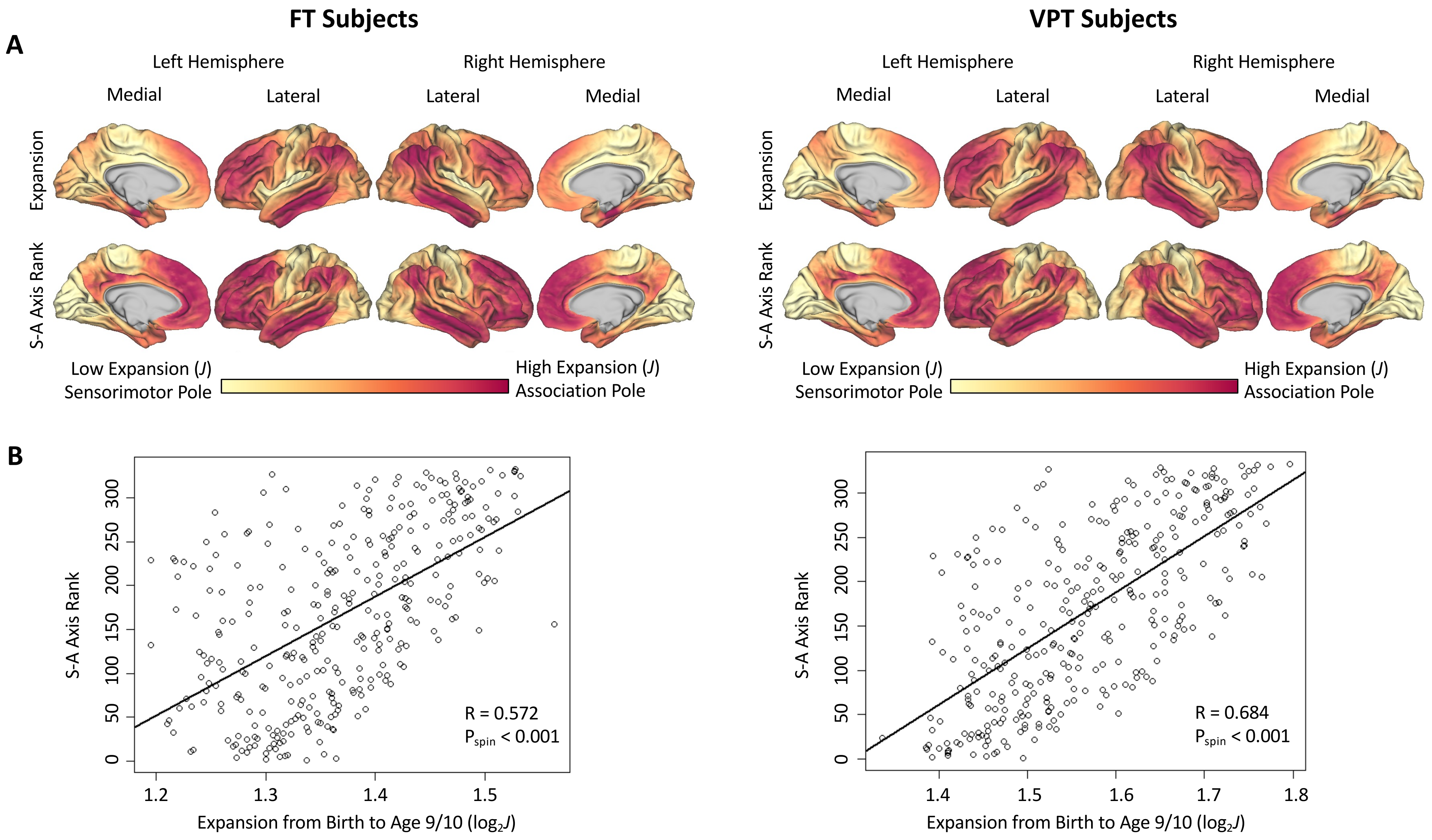

Supplementary Figure 2 Both full-term (FT) and very preterm (VPT) subjects experience expansion in association cortices. **(A)** Top: Average expansion maps, separated into FT (N=41, left) and VPT (N=52, right) groups. Bottom: Sensorimotor to association axis as described in Sydnor 2021[1], with the yellow areas representing key sensorimotor cortices and the maroon areas representing key association cortices. In all cases, color bar extremes span the 2nd to 98th percentile of individual vertex values. **(B)** For FT and VPT groups, average expansion within each Gordon 333 parcel was correlated with the sensorimotor-association axis using permutation spin tests (10,000 rotations). Log-normalized expansion is shown on the x-axis, such that a value of 1.0 represents the surface area doubling, and an expansion value of 2.0 represents the surface area quadrupling.

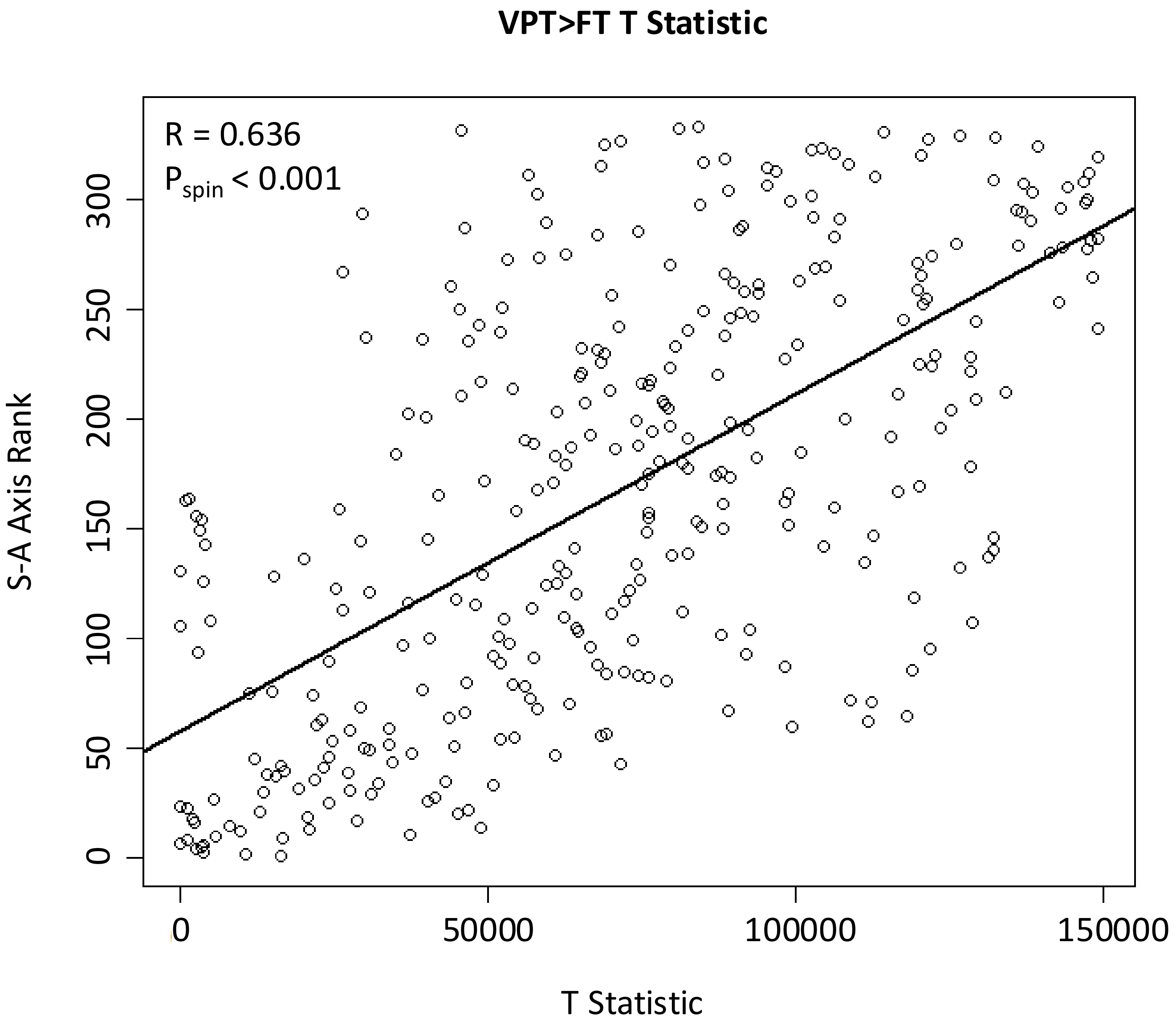

Supplementary Figure 3 Very preterm (VPT) subjects experience increased expansion in association cortices. Correlation between T statistics from the group comparison of VPT > full-term (FT) (controlling for post menstrual age (PMA) at infant scan, area deprivation index (ADI) percentile at birth, sex, and age at 9/10 scan) and sensorimotor-association (S-A) axis rank was assessed via permutation spin test. Each dot represents a region of interest (ROI) from the Gordon 333 parcellation. Higher S-A axis rank represents key association areas, and lower S-A axis rank represents key sensorimotor areas.

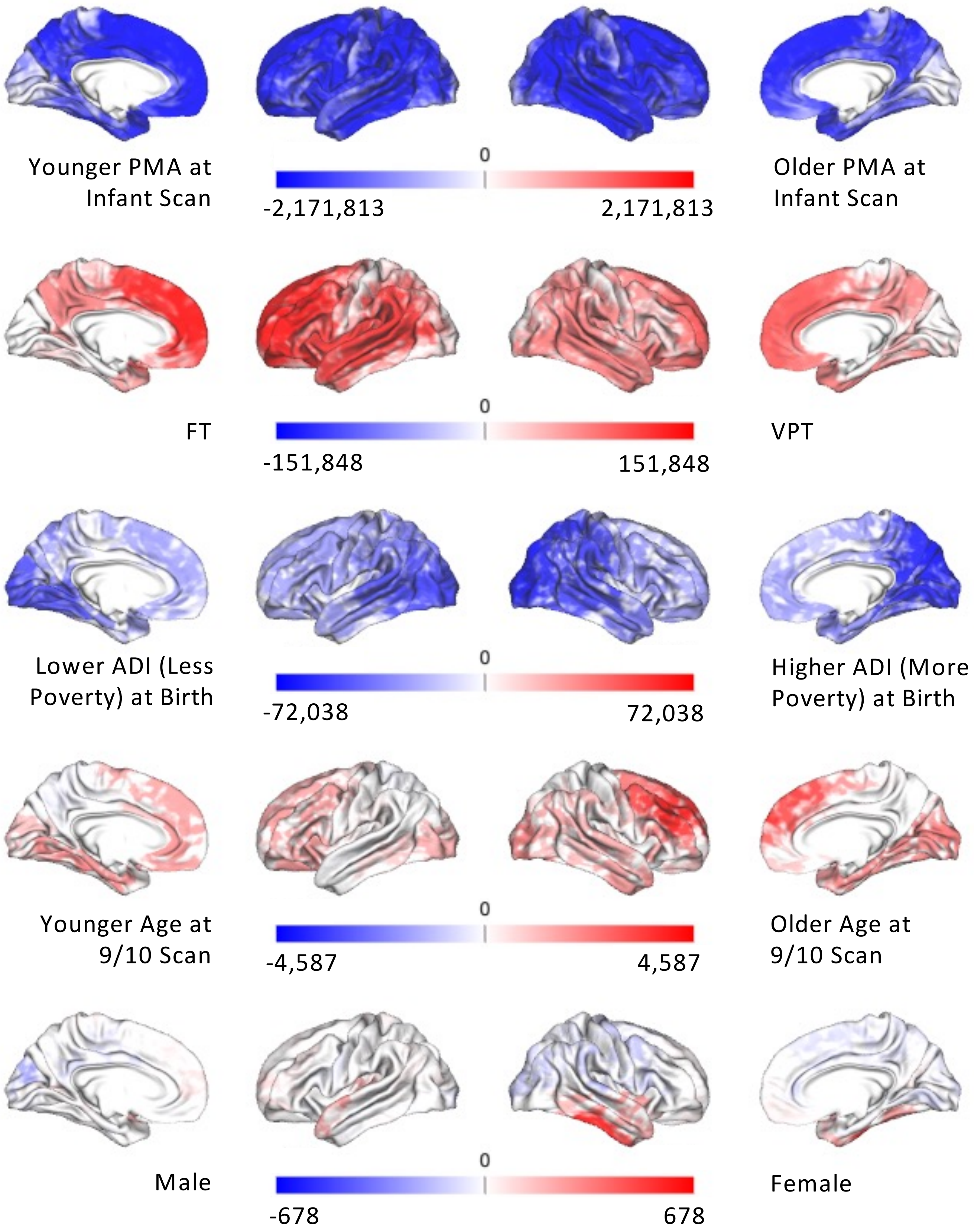

Supplementary Figure 4 Covariate effects on greater expansion. T statistics corresponding to covariate effects and main comparison of interest (very preterm (VPT) versus full-term (FT)) are reported for the two-tailed T test described in Figure 3. Effects are listed from largest to smallest, with darker blue or red suggesting an increase in cortical expansion under the listed condition. Not all colored areas pass thresholds for statistical significance.

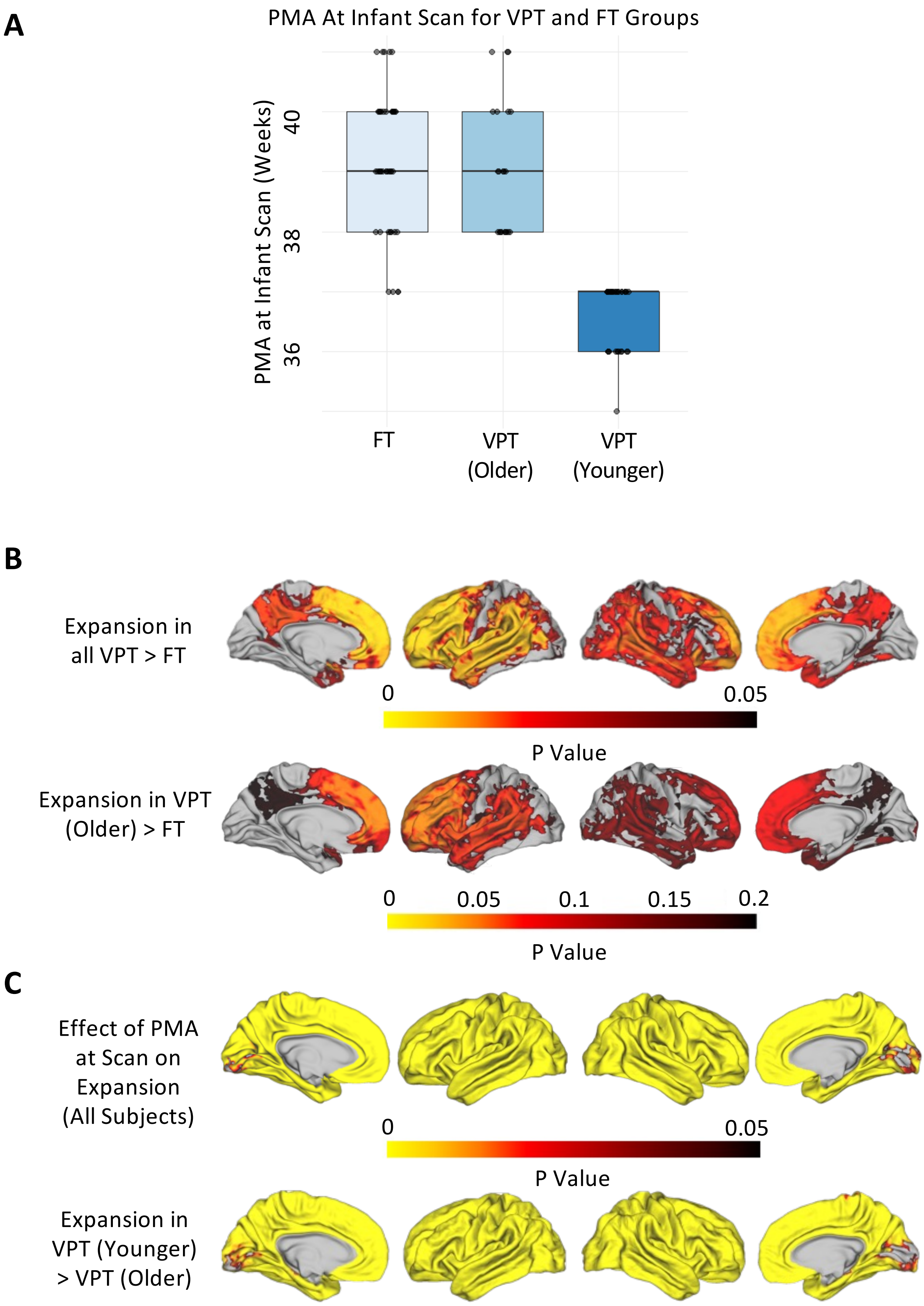

Supplementary Figure 5 Post menstrual age (PMA) at infant scan and prematurity exert separate effects. (A) Boxplot showing PMA at infant scan. Subjects are grouped into full-term (FT) controls (N=41), very preterm (VPT) subjects scanned at an older PMA (> 37 weeks)

(N=21), and VPT subjects scanned at a younger PMA (≤ 37 weeks) (N= 31). Each dot represents an individual subject. VPT (older) and FT subjects are matched in terms of mean PMA at infant scan; this was confirmed using a two-tailed T test ($p > 0.05$). **(B)** The top row shows areas where VPT children had significantly greater expansion than the FT controls when controlling for PMA at infant scan only. The bottom row shows areas where the VPT children scanned at an older PMA have greater expansion compared to the FT controls, omitting covariate effects. This parallels the top row effect, although power is lower due to the smaller VPT group matched on PMA at infant scan. **(C)** The top row illustrates regions where younger PMA at infant scan was significantly associated with increased expansion over the ten-year period (all subjects are included) when controlling for prematurity only. The second row illustrates regions where the VPT children scanned at a younger PMA have significantly greater expansion compared to the VPT children scanned at an older PMA, omitting covariate effects.

Medical Characteristics of VPT Subjects: N (%)	
Prolonged Oxygen Supplementation	27 (52%)
Patent Ductus Arteriosus	22 (42%)
Necrotizing Enterocolitis	3 (6%)
Received Postnatal Steroids	7 (13%)
Confirmed Sepsis	13 (25%)
Did Not Receive Antenatal Steroids	5 (10%)
Upper Quartile for Total Parental Nutrition	10 (19%)
Retinopathy of Prematurity Surgery	8 (15%)
Intrauterine Growth Restriction	3 (6%)
$\geq$3 SD Decrease in Weight for Height/Length from Birth to Term-Equivalent Age	0 (0%)

Supplementary Table 1 Demographic characteristics of the VPT subjects. Data is displayed as N, followed by percentage of the sample. Of note, 5 subjects were missing data for "did not receive antenatal steroids," "upper quartile for total parental nutrition," and "intrauterine growth restriction."

Relative Expansion from Infancy to Age 9/10						
	Left Hemisphere Expansion			Right Hemisphere Expansion		
Variable	Estimate	T Value	P Value	Estimate	T Value	P Value
Prematurity	0.189	2.772	0.007**	0.192	2.591	0.011*
ADI National Percentile at Birth	-0.002	-1.935	0.056	-0.003	-1.951	0.054
Age at infant scan (PMA in weeks)	-0.134	-6.134	<0.001***	-0.137	-5.738	<0.001***
Sex (Female)	-0.006	-0.106	0.916	-0.007	-0.108	0.914
Age at 9/10 scan	0.010	0.257	0.798	0.022	0.493	0.623

Supplementary Table 2 Relative expansion from infancy to age 9/10. Relative expansion for each hemisphere was calculated by dividing total hemispheric surface area at age 9/10 by total hemispheric surface area at term-equivalent age for each individual. Next, linear models were used to examine the relationships between prematurity, ADI percentile, age at scan, and sex with hemispheric surface area expansion over the ten-year period. Results paralleled our spatial findings with PALM. Both models (left and right hemisphere) have 87 degrees of freedom. * indicates P < 0.025; ** indicates P < 0.01; *** indicates P < 0.001.

Surface Area at Term-Equivalent Age						
	Left Hemisphere			Right Hemisphere		
Variable	Estimate	T Value	P Value	Estimate	T Value	P Value
Prematurity	-2372.83	-3.406	0.001**	-2426.98	-3.504	<0.001***
ADI National Percentile at Birth	-42.50	-3.054	0.003**	-33.46	-2.419	0.018*
Age at infant scan (PMA in weeks)	1565.42	6.794	<0.001***	1554.55	6.787	<0.001***
Sex (Female)	-2207.33	-3.612	<0.001***	-2127.47	-3.501	<0.001***
**Surface Area at Age 9/10 Years**						
	Left Hemisphere			Right Hemisphere		
Variable	Estimate	T Value	P Value	Estimate	T Value	P Value
Prematurity	-2886.94	-1.672	0.099	-3056.56	-1.771	0.081
ADI National Percentile at 9/10	-143.96	-3.771	<0.001***	-137.81	-3.611	<0.001***
Age at 9/10 scan (Years)	-700.54	-0.592	0.556	-446.49	-0.377	0.707
Sex (Female)	-6229.18	-3.476	<0.001***	-6486.13	-3.620	<0.001***

Supplementary Table 3 Cross-sectional results in a neighborhood-disadvantage-matched sample. A smaller sample (N= 41 VPT, 41 FT) matched by ADI percentile at age 9/10 across the VPT/FT groups was created, and linear models were conducted at both the infant and age 9/10 time points to examine the effects of our key variables of interest on surface area. Each of the four models (left and right hemispheres; TEA and age 9/10) have 77 degrees of freedom. * indicates P < 0.025; ** indicates P < 0.01; *** indicates P < 0.001.

Surface Area at Term-Equivalent Age						
	Left Hemisphere			Right Hemisphere		
Variable	Estimate	T Value	P Value	Estimate	T Value	P Value
Prematurity	-2870.13	-4.145	<0.001***	-2887.16	-4.170	<0.001***
ADI National Percentile at Birth	-30.70	-2.374	0.020*	-27.14	-2.099	0.039
Age at infant scan (PMA in weeks)	1483.96	6.467	<0.001***	1495.41	6.518	<0.001***
Sex (Female)	-2117.11	-3.437	<0.001***	-2171.56	-3.527	<0.001***
**Surface Area at Age 9/10 Years**						
	Left Hemisphere			Right Hemisphere		
Variable	Estimate	T Value	P Value	Estimate	T Value	P Value
Prematurity	-2330.96	-1.327	0.188	-2463.99	-1.396	0.167
ADI National Percentile at 9/10	-162.94	-4.758	<0.001***	-161.50	-4.692	<0.001***
Age at 9/10 scan (Years)	-714.77	-0.606	0.546	-508.70	-0.429	0.669
Sex (Female)	-5739.05	-3.233	0.002**	-6001.60	-3.364	0.001**

Supplementary Table 4 Cross-sectional results in a sample without multiples. A smaller sample (N= 44 VPT, 41 FT) was created with twins/triplets removed, so that only one sibling from each pair/trio remained in the sample. Linear models were conducted at both the infant and age 9/10 time points in order to examine the effects of our key variables of interest on surface area. Each of the four models (left and right hemispheres; TEA and age 9/10) have 80 degrees of freedom. * indicates P < 0.025; ** indicates P < 0.01; *** indicates P < 0.001.

Surface Area at Term-Equivalent Age						
	Left Hemisphere			Right Hemisphere		
Variable	Estimate	T Value	P Value	Estimate	T Value	P Value
Prematurity	-2896.08	-4.114	<0.001***	-2918.96	-4.182	<0.001***
ADI National Percentile at Birth	-28.52	-2.057	0.043	-23.21	-1.688	0.095
Age at infant scan (PMA in weeks)	1482.13	6.303	<0.001***	1483.39	6.362	<0.001***
Sex (Female)	-2152.24	-3.383	0.001**	-2140.86	-3.394	0.001**
**Surface Area at Age 9/10 Years**						
	Left Hemisphere			Right Hemisphere		
Variable	Estimate	T Value	P Value	Estimate	T Value	P Value
Prematurity	-2493.50	-1.397	0.166	-2681.14	-1.503	0.137
ADI National Percentile at 9/10	-151.03	-4.031	<0.001***	-144.99	-3.871	<0.001***
Age at 9/10 scan (Years)	-672.73	-0.562	0.576	-444.47	-0.371	0.711
Sex (Female)	-5881.14	-3.217	0.002**	-6145.49	-3.364	0.001**

Supplementary Table 5 Cross-sectional results in a neighborhood-disadvantage-matched sample without multiples. A smaller sample (N= 41 VPT, 41 FT) was created with twins/triplets removed, so that only one sibling from each pair/trio remained in the sample, and then this sample was further refined so that the VPT and FT groups were matched in terms of ADI percentile at age 9/10. Linear models were conducted at both the infant and age 9/10 time points to examine the effects of our key variables of interest on surface area. Each of the four models (left and right hemispheres; TEA and age 9/10) have 77 degrees of freedom. * indicates P < 0.025; ** indicates P < 0.01; *** indicates P < 0.001.

Total Cortical Gray Matter Volume at Term-Equivalent Age			
**Variable**	**Estimate**	**T Value**	**P Value**
Prematurity	-4689.85	-2.138	0.035*
ADI National Percentile at Birth	-78.80	-1.961	0.053
PMA at infant scan	5000.27	7.105	<0.001***
Sex (Female)	-6031.42	-3.183	0.002**

Total Cortical Gray Matter Volume at Age 9/10			
**Variable**	**Estimate**	**T Value**	**P Value**
Prematurity	-18897.3	-1.974	0.052
ADI National Percentile at 9/10	-1219.7	-6.683	<0.001***
Age at 9/10 scan	-6504.4	-0.990	0.325
Sex (Female)	-38316.2	-4.061	<0.001***

Supplementary Table 6 Cross-sectional gray matter results. Total cortical gray matter volume in infancy and at age 9/10 predicted by prematurity, ADI percentile, age at scan, and sex. Each of the models (TEA and age 9/10) have 88 degrees of freedom. * indicates P < 0.05; ** indicates P < 0.01; *** indicates P < 0.001.

Total Cerebral White Matter Volume at Term-Equivalent Age			
**Variable**	**Estimate**	**T Value**	**P Value**
Prematurity	-6127.12	-1.892	0.062
ADI National Percentile at Birth	-170.18	-2.868	0.005**
PMA at infant scan	3538.41	3.406	<0.001***
Sex (Female)	-10542.06	-3.769	<0.001***
**Total Cerebral White Matter Volume at Age 9/10**			
**Variable**	**Estimate**	**T Value**	**P Value**
Prematurity	-39932.6	-3.687	<0.001***
ADI National Percentile at 9/10	-975.0	-4.722	<0.001***
Age at 9/10 scan	5755.8	0.774	0.441
Sex (Female)	-42653.9	-3.995	<0.001***

Supplementary Table 7 Cross-sectional white matter results. Total cerebral white matter volume in infancy and at age 9/10 predicted by prematurity, ADI percentile, age at scan, and sex. Each of the models (TEA and age 9/10) have 88 degrees of freedom. * indicates P < 0.05; ** indicates P < 0.01; *** indicates P < 0.001.

References

1. Sydnor VJ, Larsen B, Bassett DS, et al. Neurodevelopment of the association cortices: Patterns, mechanisms, and implications for psychopathology. *Neuron*. Sep 15 2021;109(18):2820-2846. doi:10.1016/j.neuron.2021.06.016
